# Traumatic pericardial rupture with skeletonized phrenic nerve

**DOI:** 10.1186/1749-8090-6-6

**Published:** 2011-01-17

**Authors:** Zain Khalpey, Taufiek K Rajab, Jan D Schmitto, Philipp C Camp

**Affiliations:** 1 Division of Cardiac Surgery, Brigham and Women's Hospital, Harvard Medical School, Boston, USA; 2 Department of Cardiac, Thoracic, Transplantation and Vascular Surgery, Hannover Medical School, Hannover, Germany

## Abstract

**Background:**

Traumatic pericardial rupture is a rare presentation. Pericardial rupture itself is asymptomatic unless complicated by either hemorrhage or herniation of the heart through the defect. Following diagnosis surgical repair of the pericardium is indicated because cardiac herniation may result in vascular collapse and sudden death.

**Objectives:**

Here we present a case of traumatic, non-herniated pericardial rupture with complete skeletonization of the phrenic nerve.

**Case report:**

An 18-year-old healthy male suffered multi-trauma after falling 50 feet onto concrete. The patient could not be stabilized despite exploratory laparotomy with splenectomy, IR embolization and packing for a liver laceration. Right posterolateral thoracotomy revealed a ruptured pericardium with a completely skeletonized phrenic nerve. The pericardium was repaired with a Goretex(R) patch.

**Conclusion:**

A high level of suspicion for pericardial rupture is necessary in all patients with high-velocity thoracic injuries.

## Background

Traumatic pericardial rupture is a rare presentation. Among 20,000 patients admitted to a major trauma center only 22 were found to have blunt traumatic pericardial rupture [1]. Non-penetrating pericardial rupture most commonly results from deceleration injury [1]. In an autopsy study of 546 consecutive patients with nonpenetrating cardiac trauma, the incidence of isolated pericardial rupture was 3% [2]. Here we present a case of traumatic, non-herniated pericardial rupture with complete skeletonization of the phrenic nerve.

## Case presentation

**Figure 1:**
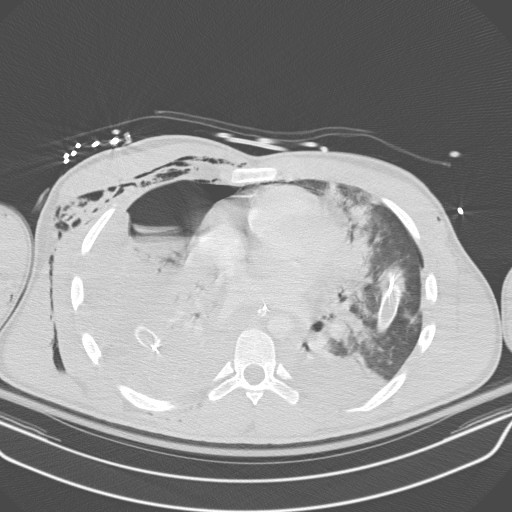
Contrast CT chest showed pneumopericardium, pneumomediastinum, right hydropneumothorax and subcutaneous emphysema.

**Figure 2:**
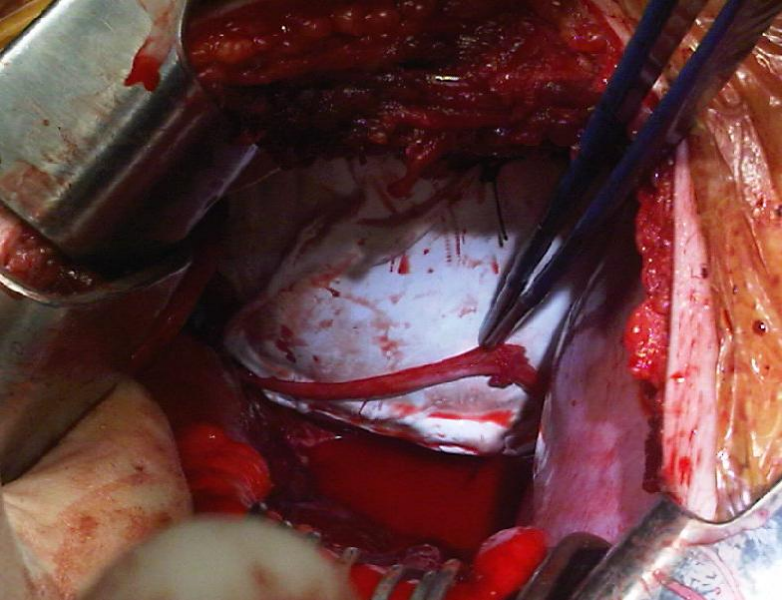
**Goretex^® ^patch repair of ruptured right pericardium. **The phrenic nerve is seen

An 18-year-old healthy male fell 50 feet onto concrete. Following resuscitation and intubation in the field, a right-sided tension pneumothorax was relieved by needle decompression. The primary survey revealed right chest dullness to percussion with decreased breath-sounds as well as upper extremity bone fractures. Chest x-ray indicated pneumomediastinum, subcutaneous emphysema, right lung opacification and rib fractures. A right-sided chest tube evacuated 500 ml blood. Non-contrast head CT showed no acute intracranial injury but an abdominal ultrasound revealed free fluid in Morison's pouch. The patient could not be stabilized despite exploratory laparotomy with splenectomy, IR embolization and packing for a liver laceration. Contrast enhanced chest CT at the time of emobolization indicated a pneumopericardium and right hemothorax (Figure 1). Right posterolateral thoracotomy revealed a ruptured pericardium extending from the diaphragm to the superior vena cava. The phrenic nerve was skeletonized but intact. Bleeding from the phrenic artery and the 9th intercostal artery was controlled by ligation. The pericardium was repaired with a Goretex^® ^patch (Figure 2). Post-operatively, the patient stabilized and made an uncomplicated recovery. Follow-up chest x-rays demonstrated normal cardiopulmonary and diaphragmatic silhouettes.

## Conclusion

Anatomically, the phrenic nerve is contained within the pericardiophrenic neurovascular bundle, which comprises the nerve, pericardiophrenic artery, and pericardiophrenic vein. This structure, together with its surrounding fat pad offers some protection to the nerve during pericardial rupture. Pericardial rupture itself is asymptomatic unless complicated by either hemorrhage or herniation of the heart through the defect. Physical examination may reveal a characteristic murmur produced by the heart beating in a hemo-pneumopericardium [3]. Radiological investigations provide additional diagnostic information but a definitive diagnosis is usually only made intra-operatively. Surgical repair is indicated because cardiac herniation may result in vascular collapse and sudden death. A high level of suspicion for pericardial rupture is necessary in all patients with high-velocity thoracic injuries.

## Consent

Informed consent was obtained from the patient for publication of this case report and any accompanying images.

## Competing interests

The authors declare that they have no competing interests.

## Authors' contributions

ZK and PCC were involved in patient care. TKR and JDS reviewed the literature, wrote the manuscript. PCC supervised the study. All authors read and approved the final manuscript.
